# Engagement and Weight Change in an Online Weight Management Program in Clinical Settings: An Implementation Project

**DOI:** 10.1007/s11606-026-10493-5

**Published:** 2026-06-03

**Authors:** Heather J. Baer, JoAnn Cho, Richard Fay, Caroline M. Apovian, Ashley C. McCarthy, Alexander J. Blood, Lipika Samal, Naomi D. L. Fisher, E. John Orav, Matthew Wien, Jorge Plutzky, Jason P. Block, David W. Bates, Ronen Rozenblum, Michela Tucci, Marian McPartlin, Michael Oates, Katherine D. McManus, Benjamin M. Scirica

**Affiliations:** 1 Division of General Internal Medicine and Primary Care, Brigham and Women’s Hospital, Boston, MA USA; 2 Harvard Medical School, Boston, MA USA; 3 Harvard T.H. Chan School of Public Health, Boston, MA USA; 4 Division of Endocrinology, Diabetes and Hypertension, Brigham and Women’s Hospital, Boston, MA USA; 5 Division of Cardiovascular Medicine, Department of Medicine, Brigham and Women’s Hospital, Boston, MA USA; 6 Department of Population Medicine, Harvard Pilgrim Health Care Institute, Harvard Medical School, Boston, MA USA; 7 Accelerator for Clinical Transformation, Mass General Brigham, Boston, MA USA; 8 Department of Nutrition, Brigham and Women’s Hospital, Boston, MA USA

**Keywords:** overweight, obesity, online, coaching, implementation

## Abstract

**BACKGROUND:**

There is an urgent need for scalable strategies for treatment of overweight and obesity that can be implemented in clinical settings.

**OBJECTIVE:**

To implement and evaluate an online weight management program in a large, diverse population of patients.

**DESIGN:**

Clinical implementation project in primary care and specialty clinics.

**PARTICIPANTS:**

Eligible patients were ≥ 20 years old, spoke English or Spanish, and had a body mass index (BMI) of ≥ 30 kg/m^2^ or a BMI of 25–29.9 kg/m^2^ plus ≥ 1 cardiovascular risk factor or obesity-related condition.

**INTERVENTION:**

Enrolled patients were encouraged to register for a 12-month digital health program called RestoreHealth, which included an online program/app and coaching.

**MAIN MEASURES:**

We examined recruitment and enrollment in the program, as well as engagement, absolute and percent weight change, and predictors of weight change during the first six months. Subgroup analyses were conducted by use of anti-obesity medications.

**KEY RESULTS:**

A total of 5056 patients enrolled between November 2022 and October 2023, and 4511 (89.2%) completed registration for RestoreHealth. At six months, mean absolute and percent weight change among all enrolled patients were -5.7 pounds and -2.5%, respectively (p < 0.0001 for both), and 27.7% of patients lost ≥ 5% of their body weight. There was greater weight loss among patients using anti-obesity medications (mean absolute and percent weight change = -12.5 pounds and -5.5%, respectively). Among registered patients, 91.8% engaged in the program, although engagement decreased over time. Older age, greater weight at enrollment, and longer duration of engagement were associated with greater weight loss.

**CONCLUSIONS:**

We rapidly enrolled a large, diverse population of patients in PROPS 2.0. Patients lost weight at six months, with greater weight loss among those using anti-obesity medications. This program has the potential to be scaled and implemented at other institutions in the future.

**ClinicalTrials.gov registration number:**

NCT05553925 (initial release date August 18, 2022).

**Supplementary Information:**

The online version contains supplementary material available at https://doi.org/10.1007/s11606-026-10493-5.

## INTRODUCTION

Almost 75% of U.S. adults have overweight (body mass index [BMI] 25–29.9 9 kg/m^2^) or obesity (BMI ≥ 30 kg/m^2^),^1^ which are associated with increased morbidity and mortality from many chronic conditions.^2^ Clinical guidelines recommend comprehensive lifestyle intervention for all patients with obesity and for patients with overweight who have cardiovascular risk factors or other obesity-related conditions.^3,4^ However, clinicians often are not able to counsel patients about weight due to limited time, training, and resources.^5–7^ There is a need for scalable and sustainable strategies for treatment of overweight and obesity that can be implemented in clinical settings. Even with increasing use of anti-obesity medications such as glucagon-like peptide-1 receptor agonists (GLP-1s), lifestyle intervention is still the first-line treatment for overweight and obesity and is always recommended as an adjunct to pharmacotherapy.^4,8^

Online programs that deliver comprehensive lifestyle intervention are effective and may be easier to access than in-person programs, although the magnitude of weight loss may be less.^9–11^ Despite evidence supporting online weight management programs, relatively few studies have focused on evaluating their pragmatic implementation in routine clinical settings.^12,13^ Our team previously completed a pragmatic, cluster-randomized trial of an online program in primary care, called the PROPS Study; this study demonstrated significantly greater weight loss at 12 months among patients randomized to an online program integrated with population health management than among those randomized to an online program alone or to usual care.^14,15^

The current project, PROPS 2.0, was a clinical implementation project that used an existing online program and adapted the intervention from the original PROPS Study, in order to implement and evaluate it in a larger, more diverse population. The purpose of this paper is to describe recruitment and enrollment in PROPS 2.0, including measures of reach and adoption, as well to examine engagement, weight change, and predictors of weight change during the first six months of enrollment.

## METHODS

### Design and Setting

PROPS 2.0 was a 12-month program implemented as part of standard care (at no cost to patients) in general medicine and specialty clinics (Primary Care, Cardiology, Endocrinology, Orthopedic Surgery, Comprehensive Breast Health, Sleep Medicine, and the Center for Weight Management and Wellness) affiliated with Brigham and Women’s Hospital (BWH), a large, academic medical center in Boston, Massachusetts. The design and methods, including adaptations to the intervention from the original PROPS Study, have been described in detail elsewhere.^16^

Although PROPS 2.0 was a clinical implementation project, some components were considered research. The Mass General Brigham Human Research Committee approved the project with a waiver of individual-level informed consent, and the program was registered with ClinicalTrials.gov (NCT05553925).

### Population

PROPS 2.0 was offered to all eligible patients at participating clinics who were ≥ 20 years old, had a BMI of ≥ 30 kg/m^2^ or a BMI of 25–29.9 kg/m^2^ with at least one cardiovascular risk factor or obesity-related condition, had a primary care clinician, and had received care from a BWH clinician within the last two years. Patients also had to speak English or Spanish, have a valid email address, and have regular Internet access. Patients were excluded if they were pregnant, had type 1 diabetes or were taking insulin, or had active cancer or were being treated for cancer. Patients who had weight loss surgery within the last 12 months were excluded, but those participating in other weight loss programs and/or taking anti-obesity medications were eligible.

### Recruitment and Enrollment

Patients were recruited through three pathways: 1) referral by clinicians, 2) self-referral, and 3) identification via the electronic health record (EHR). For the clinician referral pathway, an internal referral order was created in Epic, the EHR at BWH. In addition, two Best Practice Advisories (BPAs) were developed in Epic to alert clinicians about patients who were potentially eligible. If one of the BPAs appeared at the time of a patient’s visit, the clinician could directly place the referral order through the BPA. After patients were referred, their information was imported into a customized customer relationship management (CRM) platform,^17^ which was used for tracking and outreach. Patients would then receive an electronic message describing the program and asking them to complete a brief enrollment survey to verify their eligibility. We educated clinicians and other staff about the program through presentations and pocket-sized cards with information about the program and referral process.

The self-referral pathway utilized a website with information about the program for patients. Patients could access the enrollment survey from the website to verify their eligibility, and their information was then imported into the CRM platform. We distributed posters and business cards for patients at all participating clinics, with a QR code and link to the website.

The EHR identification pathway was used to enhance recruitment for two specific groups of patients: Spanish-speaking patients, and patients who had an initial visit scheduled at the Center for Weight Management and Wellness with a wait time of over three months. We extracted information for these patients from the EHR and imported it into the CRM platform, and we sent outreach messages (including a link to the enrollment survey) to patients who appeared to meet eligibility criteria, following a similar workflow as the clinician referral pathway.

### Online Program/App (RestoreHealth) and Patient Navigator Support

Patients who were eligible and decided to enroll received a link to register for RestoreHealth (HealthFleet, Inc., Needham MA, https://www.healthfleet.com). RestoreHealth is a 12-month digital health program that pairs an online program/app with one-on-one coaching. The program focuses on nutrition, exercise, sleep, and stress and is designed to support behavior modification via easy and simple interactions and personalized coaching. It is available in English and Spanish and has separate interfaces for patients and coaches.

After patients registered for RestoreHealth, they could view brief lessons, log meals, and get personalized feedback from an assigned coach. RestoreHealth also integrates data from digital scales and sleep/activity trackers, which can be connected to the platform, allowing patients’ data to be uploaded automatically. These devices were initially provided to patients as part of the program; however, RestoreHealth ended their provision of them during the enrollment period, so patients who enrolled later did not receive them. The coaches were behavioral change specialists employed by RestoreHealth and certified through the National Board for Health and Wellness Coaching. Coaches monitored patients’ data and gave personalized feedback. They primarily corresponded with patients via text messaging but scheduled monthly phone calls with patients who preferred this. Communication was tailored based on patients’ preferences, but coaches typically decreased the frequency of text messages or calls after the first four or five months.

Patient navigators, who were non-clinical staff members from Mass General Brigham, helped with enrollment and provided some administrative support during the program. In particular, the navigators sent outreach messages about enrollment to referred patients using the CRM platform, entered documentation into the EHR, and monitored weight data to identify and conduct outreach to patients with rapid weight loss.^16^

### Data Collection and Follow-Up

After patients completed registration for RestoreHealth, they participated in the program for 12 months. There were no in-person visits, other than visits with their clinicians as part of their usual care. Engagement data were collected within the online program/app, as well as from connected devices. Data on demographic and clinical variables, including medical conditions and use of anti-obesity medications, were extracted from the EHR and assessed by self-report on the enrollment survey. We defined four categories of anti-obesity medication use based on the timing of prescriptions in the EHR: 1) no use within 7 months before or after enrollment; 2) use within 7 months before but not after enrollment; 3) use within 7 months after but not before enrollment; and 4) use within 7 months both before enrollment and after enrollment.

### Outcomes

The full list of outcomes has been described previously.^16^ For this analysis, we focused on measures of program reach (e.g., number of patients referred or identified, enrolled, registered), as well as weight change and engagement during the first six months after enrollment. We calculated absolute weight change as the difference between each patient’s initial weight at RestoreHealth registration (or at enrollment if they did not complete registration) and their weight approximately six months later. Weights were obtained from digital scales provided by RestoreHealth, the EHR, or self-report (only at enrollment if no other available weight). Hierarchical preferences were given to baseline (42 days before to 30 days after registration) and six-month (± 60 days) weights from the same source, with digital scales having priority over EHR, and both over self-report (Supplement eTable 1). We chose ≤ 42 days before registration for baseline weight to be consistent with the original PROPS trial.^15^ We also calculated percent weight change and a binary indicator of ≥ 5% weight loss at six months.

We examined engagement in each individual component of the program as well as in three categories: 1) engagement with the coach (text messages and calls), 2) engagement with the app (viewing lessons, logging meals, completing exercise or other activities), and 3) engagement with devices (using digital scales and sleep/activity trackers). For each component, the patient was counted as engaged if they had at least one of that type during the first six months of enrollment. We also calculated engagement by program month and total number of months of engagement per patient. For engagement by month, the patient was counted as engaged in that month if they had any type of engagement during their first month (i.e., 30 days) of the program, second month, etc. For total months of engagement, we summed the number of months when each patient had any type of engagement.

### Statistical Analysis

We examined descriptive characteristics (means and standard deviations [SD] for continuous variables and percentages for binary and categorical variables) for all enrolled patients and by registration status, and we used standardized mean differences (SMDs) to compare registered and non-registered patients. We then examined six-month weight change (means and 95% confidence intervals [CI]) among all enrolled patients, using linear regression models for absolute and percent weight change and a logistic regression model for ≥ 5% weight loss, adjusting for clustering due to referring clinician using generalized estimating equations. Since this was an implementation project and not a randomized trial, we used patients who enrolled in the program but did not register for RestoreHealth as a quasi-control group. We compared six-month weight change between patients who completed registration and those who did not, adjusting for clinician and pre-specified covariates, including age, sex, race, ethnicity, primary language, weight at enrollment, medical conditions at enrollment, and use of anti-obesity medications. We also conducted subgroup analyses by use of anti-obesity medications.

Multiple imputation with chained equations was used to ensure that weight changes could be based on weights either measured or imputed from the same source (Supplement eTable 1). The imputation model included all outcome variables, registration status, and all patient characteristics in Table 1. Modelling results reflect the aggregation across 25 imputations. Descriptive results are based on the actual data.

We calculated the number and percentage of patients with each individual type and each category of engagement. In addition, we examined the associations of individual engagement variables with absolute six-month weight change in univariable linear regression models. We also used mixed effects linear regression models to examine patient characteristics associated with six-month weight change, using the same covariates mentioned previously. Finally, we added engagement measures to these models to examine them as potential mediators of weight change. A p value of < 0.05 was used for statistical significance; since these were not protocol-specified analyses, we did not adjust for multiple comparisons. All analyses were conducted using SAS version 9.4 (SAS Institute Inc).

## RESULTS

### Participants

**Figure 1:**
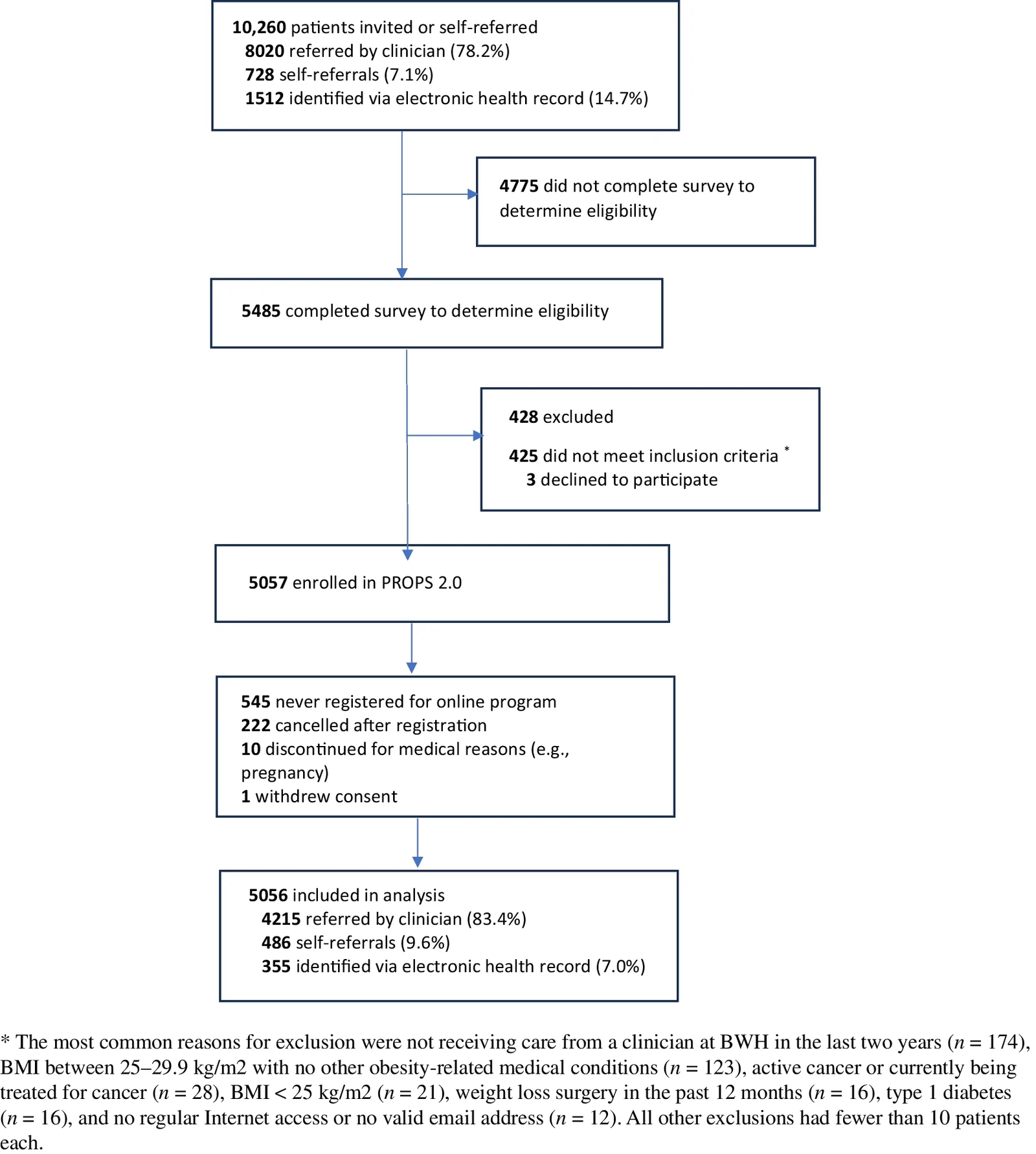
Flow of participants

From November 2022 through October 2023, a total of 10,260 patients were referred, self-referred, or invited to participate in the program; 8020 (78.2%) were referred by 441 clinicians (approximately 65% of clinicians at participating clinics), 728 self-referred (7.1%), and 1512 were identified via the EHR (14.7%) (Fig. 1). Among these patients, 5485 (52.7%) completed the enrollment survey to assess eligibility, and 5057 (92.2%) were eligible and enrolled; monthly enrollment by referral source is shown in Supplement eFigure 1. One patient withdrew consent after registration; therefore, 5056 patients were included in the analysis. Of these, 4511 patients (89.2%) completed registration for RestoreHealth,

At enrollment, patients’ mean age was 50.5 years (SD = 14.0), and mean BMI was 36.0 kg/m^2^ (SD = 6.4); 77.5% of patients were female, 73.6% were white, 13.9% were Hispanic, 93.8% spoke English as their primary language, 84.0% had private insurance, and 47.8% completed college as their highest level of education (Table 1). The most common medical conditions were prediabetes or insulin resistance (29.9%), hypertension (45.2%), and hyperlipidemia (43.8%), and 18.5% of patients were using anti-obesity medications before and after enrollment. Some characteristics were different for patients who completed registration and those who did not, with SMDs greater than 0.10; for example, a higher percentage of registered patients were white, non-Hispanic, English-speaking, and college-educated, and a higher percentage of patients who did not register had type 2 diabetes.

**Table 1:** Demographic and Clinical Characteristics of Enrolled Patients, Overall and by Registration Status

	All enrolled (*n* = 5056)	Registered (*n* = 4511)	Not registered (*n* = 545)	SMD
Characteristic	Mean (SD)	Mean (SD)	Mean (SD)	
Age, years	50.5 (14.0)	50.4 (14.0)	51.8 (14.1)	0.10
Body mass index (BM), kg/m^2^	36.0 (6.4)	36.1 (6.5)	35.6 (5.7)	0.08
	**No. (%)**	**No. (%)**	**No. (%)**	
Age (years)				
< 40	1213 (24.0)	1100 (24.4)	113 (20.7)	0.09
40–49	1029 (20.4)	926 (20.5)	103 (18.9)	0.04
50–59	1360 (26.9)	1210 (26.8)	150 (27.5)	0.02
60–69	997 (19.7)	868 (19.2)	129 (23.7)	0.11
≥ 70	457 (9.0)	407 (9.0)	50 (9.2)	0.01
Sex				
Female	3920 (77.5)	3518 (78.0)	402 (73.8)	0.10
Male	1136 (22.5)	993 (22.0)	143 (26.2)	
Race				
White	3721 (73.6)	3422 (75.9)	299 (54.9)	0.45
Black or African American	578 (11.4)	504 (11.2)	74 (13.6)	0.07
Asian or Pacific Islander	148 (2.9)	134 (3.0)	14 (2.6)	0.02
Other	465 (9.2)	339 (7.5)	126 (23.1)	0.44
Missing	144 (2.9)	112 (2.5)	32 (5.9)	0.17
Ethnicity				
Hispanic	705 (13.9)	526 (11.7)	179 (32.8)	0.53
Not Hispanic	4016 (79.4)	3683 (81.6)	333 (61.1)	0.47
Missing	335 (6.6)	302 (6.7)	33 (6.1)	0.03
Primary language				
English	4744 (93.8)	4342 (96.2)	402 (73.8)	0.66
Spanish	288 (5.7)	148 (3.3)	140 (25.7)	0.67
Other or missing	24 (0.5)	21 (0.5)	3 (0.6)	0.01
Health insurance				
Private	4248 (84.0)	3811 (84.5)	437 (80.2)	0.11
Medicare	693 (13.7)	622 (13.8)	71 (13.0)	0.02
Medicaid/other government	115 (2.3)	78 (1.7)	37 (6.8)	0.25
Highest level of education				
High school or less	1267 (25.1)	1032 (22.9)	235 (43.1)	0.44
College	2416 (47.8)	2221 (49.2)	195 (35.8)	0.28
Graduate school	728 (14.4)	677 (15.0)	51 (9.4)	0.17
Missing	645 (12.8)	581 (12.9)	64 (11.7)	0.04
Medical conditions				
Type 2 diabetes	672 (13.3)	557 (12.4)	115 (21.1)	0.24
Prediabetes or insulin resistance	1514 (29.9)	1331 (29.5)	183 (33.6)	0.09
Hypertension	2287 (45.2)	2032 (45.1)	255 (46.8)	0.04
Hyperlipidemia	2215 (43.8)	1961 (43.5)	254 (46.6)	0.06
Coronary heart disease	274 (5.4)	242 (5.4)	32 (5.9)	0.02
Stroke	72 (1.4)	65 (1.4)	7 (1.3)	0.01
Obstructive sleep apnea	1354 (26.8)	1192 (26.4)	162 (29.7)	0.07
Osteoarthritis	889 (17.6)	778 (17.3)	111 (20.4)	0.08
Non-alcoholic fatty liver disease	589 (11.7)	507 (11.2)	82 (15.1)	0.11
Polycystic ovary syndrome	312 (6.2)	283 (6.3)	29 (5.3)	0.04
Gastroesophageal reflux disease	1413 (28.0)	1255 (27.8)	158 (29.0)	0.03
Gallbladder disease	280 (5.5)	235 (5.2)	45 (8.3)	0.12
Use of anti-obesity medications^*^				
No use before or after enrollment	3160 (62.5)	2817 (62.5)	343 (62.9)	0.01
Use before but not after enrollment	278 (5.5)	243 (5.4)	35 (6.4)	0.04
Use after but not before enrollment	682 (13.5)	611 (13.5)	71 (13.0)	0.02
Use before and after enrollment	936 (18.5)	840 (18.6)	96 (17.6)	0.03

SMD = standardized mean difference (between registered and non-registered patients); SD = standard deviation

^*^ Use of anti-obesity medications before enrollment was defined as receiving a prescription in the EHR within the 7 months before enrollment, and use of anti-obesity medications after enrollment was defined as receiving a prescription in the EHR within the 7 months after enrollment

### Weight Change

Mean absolute and percent weight change from baseline to six months among all enrolled patients were −5.7 pounds (95% CI, −6.1 to −5.3 pounds) and −2.5% (95% CI, −2.7 to −2.3%), respectively (*p* < 0.0001 for both), and the percentage of enrolled patients who lost ≥ 5% body weight was 27.7% (95% CI, 26.3% to 29.2%) (Table 2). In unadjusted analyses, there was greater weight loss among patients who completed registration compared to those who did not. For example, mean absolute weight change was −5.9 pounds (95% CI, −6.6 to −5.1 pounds) among patients who registered, compared to −4.3 pounds (95% CI, −5.7 to −2.9 pounds) among patients who did not register, although the difference was attenuated and non-significant after adjustment.

**Table 2:** Weight Change at Six Months Among Enrolled Patients, Overall and By Registration Status

**Absolute weight change (pounds)**		
	**Unadjusted** mean (95% CI)^*^	*P* value^*^
All enrolled	−5.7 (−6.1, −5.3)	< 0.0001
	**Unadjusted** mean (95% CI)^*^	**Adjusted** **mean (95% CI)**^**†**^
Registered	−5.9 (−6.6, −5.1)	−5.8 (−6.3, −5.3)
Not registered	−4.3 (−5.7, −2.9)	−4.8 (−6.3, −3.3)
Difference	−1.6 (−3.0, −0.1)	−1.0 (−2.6, 0.6)
**Percent weight change (percent)**		
	**Unadjusted** mean (95% CI)^*^	*P* value^*^
All enrolled	−2.5 (−2.7, −2.3)	< 0.0001
	**Unadjusted** mean (95% CI)^*^	**Adjusted** **mean (95% CI)**^**†**^
Registered	−2.6 (−3.0, −2.3)	−2.6 (−2.8, −2.4)
Not registered	−1.9 (−2.5, −1.2)	−2.1 (−2.8, −1.4)
Difference	−0.8 (−1.5, −0.1)	−0.5 (−1.3, 0.2)
**Percent with ≥ 5% weight loss (percent)**		
	**Unadjusted** percent (95% CI)^*^	
All enrolled	27.7 (26.3, 29.2)	
	**Unadjusted** percent (95% CI)^*^	**Adjusted** **percent (95% CI)**^**†**^
Registered	28.3 (26.0, 30.7)	28.1 (26.5, 29.8)
Not registered	22.5 (17.8, 27.2)	24.2 (19.3, 29.1)
Difference	5.9 (0.9, 10.9)	4.0 (−1.3, 9.2)

CI = confidence interval

^*^Unadjusted means, 95% CI, and *P* values from linear regression models (for absolute weight change and percent weight change) or a logistic regression model (for percent with ≥ 5% weight loss), adjusting for referring clinician using generalized estimating equations. No P value is included for percent with ≥ 5% weight loss because there was no clear null hypothesis for this

^†^Adjusted means and 95% CI from linear regression models (for absolute weight change and percent weight change) or a logistic regression model (for percent with ≥ 5% weight loss), adjusting for referring clinician and age, sex, race, ethnicity, primary language, baseline weight, medical conditions, and use of anti-obesity medications

Weight loss was greater among patients using anti-obesity medications before and after enrollment (Table 3 and Supplement eTable 2). For example, mean absolute weight change and percent weight change were −12.5 pounds (95% CI, −13.5 to −11.5 pounds) and −5.5% (−5.9 to −5.0%) among enrolled patients using anti-obesity medications and −3.9 pounds (95% CI, −4.4 to −3.5 pounds) and −1.8% (−2.0 to −1.6%) among enrolled patients not using them (p < 0.0001 for both comparisons). Within both subgroups, patients who completed registration had greater weight loss than those who did not, although the differences were small and non-significant after adjustment.

**Table 3:** Weight Change at Six Months Among Enrolled Patients, Stratified by Use of Anti-Obesity Medications

	No use of anti-obesity medications before or after enrollment (*n* = 3160)		Use of anti-obesity medications before and after enrollment (*n* = 936)	
	**Absolute weight change (pounds)**		**Absolute weight change (pounds)**	
	**Unadjusted** mean (95% CI) ^*^		**Unadjusted** mean (95% CI) ^*^	
All enrolled	−3.9 (−4.4, −3.5)		−12.5 (−13.5, −11.5)	
	**Unadjusted** mean (95% CI) ^*^	**Adjusted** **mean (95% CI) **^**†**^	**Unadjusted** mean (95% CI) ^*^	**Adjusted** **mean (95% CI) **^**†**^
Registered	−4.0 (−4.6, −3.5)	−4.0 (−4.5, −3.5)	−12.9 (−14.4, −11.3)	−12.7 (−14.0, −11.4)
Not registered	−3.0 (−4.6, −1.4)	−3.2 (−5.0, −1.5)	−9.4 (−12.5, −6.3)	−10.8 (−14.1, −7.5)
Difference	−1.1 (−2.7, 0.6)	−0.8 (−2.6, 1.1)	−3.5 (−6.8, −0.1)	−1.9 (−5.3, 1.5)
	**Percent weight change (percent)**		**Percent weight change (percent)**	
	**Unadjusted** mean (95% CI) ^*^		**Unadjusted** mean (95% CI) ^*^	
All enrolled	−1.8 (−2.0, −1.6)		−5.5 (−5.9, −5.0)	
	**Unadjusted** mean (95% CI) ^*^	**Adjusted** **mean (95% CI) **^**†**^	**Unadjusted** mean (95% CI) ^*^	**Adjusted** **mean (95% CI) **^**†**^
Registered	−1.8 (−2.1, −1.6)	−1.8 (−2.1, −1.6)	−5.6 (−6.3, −5.0)	−5.6 (−6.2, −5.0)
Not registered	−1.3 (−2.1, −0.6)	−1.4 (−2.2, −0.6)	−4.1 (−5.5, −2.8)	−4.6 (−6.0, −3.2)
Difference	−0.5 (−1.3, 0.2)	−0.4 (−1.3, 0.4)	−1.5 (−3.0, −0.1)	−1.0 (−2.5, 0.5)
	**Percent with ≥ 5% weight loss (percent)**		**Percent with ≥ 5% weight loss (percent)**	
	Unadjusted percent (95% CI) ^*^		**Unadjusted** percent (95% CI) ^*^	
All enrolled	21.7 (20.0, 23.3)		49.4 (45.9, 52.8)	
	**Unadjusted** percent (95% CI) ^*^	**Adjusted percent (95% CI) **^**†**^	**Unadjusted** percent (95% CI) ^*^	**Adjusted percent (95% CI) **^**†**^
Registered	22.2 (20.4, 24.1)	22.1 (20.3, 23.9)	50.6 (46.4, 54.9)	50.3 (46.4, 54.1)
Not registered	17.4 (12.2, 22.5)	18.3 (12.6, 24.0)	38.5 (27.3, 49.6)	41.6 (29.9, 53.3)
Difference	4.8 (−0.9, 10.6)	3.8 (−2.6, 10.2)	12.2 (0.3, 24.0)	8.7 (−3.6, 21.0)

CI = confidence interval

^*^Unadjusted means and 95% CI from linear regression models (for absolute weight change and percent weight change) or a logistic regression model (for percent with ≥ 5% weight loss), adjusting for referring clinician using generalized estimating equations

^†^Adjusted means and 95% CI from linear regression models (for absolute weight change and percent weight change) or a logistic regression model (for percent with ≥ 5% weight loss), adjusting for referring clinician and age, sex, race, ethnicity, primary language, baseline weight, and medical conditions

### Engagement and Predictors of Weight Change Among Registered Patients

Among the 4511 registered patients, 4139 (91.8%) engaged in at least one aspect of the program, although engagement decreased over time, with 90.8% engaging in Month 1 but only 34.5% in Month 6 (Table 4). In general, any type of engagement was associated with greater weight loss, and longer duration of engagement was associated with greater weight loss; for each additional month of engagement, there was −0.6 pounds (95% CI, −0.8 to −0.4 pounds) greater weight loss. In an adjusted post hoc analysis, registered patients who engaged for more than three months had significantly greater weight loss than patients who did not register (p = 0.003), with mean absolute weight change of −7.2 pounds (Supplement eTable 3).

**Table 4:** Referral Source and Engagement Among Registered Patients and Associations with Absolute Weight Change at Six Months

	No. (%)	Association with absolute weight change (95% CI) ^*^
	Overall mean weight change = −5.8 pounds	
Referral source^†^		
Clinician referral	3813 (84.5)	−0.9 (−2.2, 0.3)
Self-referral	430 (9.5)	1.0 (−0.4, 2.5)
Identification via electronic health record	268 (5.9)	0.6 (−1.3, 2.5)
Sent digital scale	2134 (47.3)	−0.6 (−1.5, 0.2)
≥ 1 engagement (any type)	4139 (91.8)	−1.9 (−3.4, −0.3)
≥ 1 engagement (any type) by month^‡^		
Month 1	4097 (90.8)	−1.5 (−3.0, −0.02)
Month 2	2797 (60.2)	−1.8 (−2.7, −0.9)
Month 3	2321 (51.5)	−2.2 (−3.1, −1.4)
Month 4	2052 (45.5)	−2.2 (−3.0, −1.4)
Month 5	1789 (39.7)	−2.4 (−3.2, −1.5)
Month 6	1557 (34.5)	−2.7 (−3.6, −1.9)
≥ 1 engagement of each type		
Calls with coach	1440 (31.9)	−0.9 (−1.7, −0.02)
Texts with coach	3653 (81.0)	−1.5 (−2.6, −0.4)
Lessons	3182 (70.5)	−1.6 (−2.6, −0.6)
Meals	2838 (62.9)	−1.8 (−2.7, −0.9)
Sleeps	1526 (33.8)	−1.6 (−2.5, 0.6)
Weights (digital only)	1708 (37.9)	−1.5 (−2.4, −0.7)
Exercise activities	2664 (59.1)	−1.8 (−2.7, −0.9)
Other activities	3786 (83.9)	−1.6 (−2.9, −0.4)
Surveys	3300 (73.2)	−1.4 (−2.4, −0.4)
≥ 1 engagement in each category^§^		
With coach (calls or texts)	3667 (81.3)	−1.5 (−2.6, −0.4)
With app	3961 (87.8)	−1.7 (−3.1, −0.3)
With devices	2095 (46.4)	−1.3 (−2.2, −0.5)
	**Median (25th-75th percentile)**	Association with absolute weight change (95% CI)*
Total months of engagement (0–6)	2.0 (1.0–6.0)	−0.6 (−0.8, −0.4)

CI = confidence interval; SD = standard deviation

^*^From univariable linear regression models including each individual factor, adjusting for referring clinician using generalized estimating equations. Negative values indicate greater weight loss

^†^Comparing each referral source to all other referral sources

^‡^Comparing any engagement in each month to no engagement in that month

^§^Comparing engagement in each category to no engagement in that category. Engagement with coach includes calls or texts with coach, engagement with devices including tracking sleep and tracking weight with trackers and scales, and engagement with app includes everything else

In multivariable models, older age, greater baseline weight, and use of anti-obesity medications after enrollment were associated with greater weight loss, and black race was associated with less weight loss (Table 5). These differences changed minimally and were still significant after adjustment for engagement. Similarly, longer duration of engagement was associated with greater weight loss, even after adjustment for demographic and clinical factors.

**Table 5:** Multivariable Predictors of Absolute Weight Change at Six Months Among Registered Patients

	Before adjustment for engagement^*^		After adjustment for engagement^†^	
	Association with weight change (95% CI)	*P* value	Association with weight change (95% CI)	*P* value
	**Overall mean weight change = −5.8 pounds**			
**Demographic and clinical variables**				
Age (per 10 years)	−0.8 (−1.4, −0.2)	0.007	−0.7 (−1.3, −0.2)	0.01
Baseline weight (per 10 pounds)	−0.3 (−0.4, −0.1)	0.0002	−0.3 (−0.4, −0.1)	0.0002
Male sex (reference: female)	0.6 (−1.1, 2.2)	0.50	0.4 (−1.3, 2.0)	0.67
Race (reference: white)				
Black	2.6 (0.9, 4.4)	0.004	2.6 (0.8, 4.4)	0.005
Asian	2.2 (−1.6, 5.9)	0.26	2.1 (−1.6, 5.8)	0.26
Other	1.7 (−0.9, 4.3)	0.20	1.6 (−1.0, 4.2)	0.22
Hispanic (reference: non-Hispanic)	0.8 (−1.6, 3.1)	0.51	0.7 (−1.6, 3.1)	0.54
Spanish as primary language (reference: English)	−0.9 (−4.2, 2.4)	0.60	−1.2 (−4.6, 2.1)	0.46
Type 2 diabetes (reference: none)	1.2 (−0.4, 2.8)	0.14	1.2 (−0.4, 2.8)	0.16
Prediabetes or insulin resistance (reference: none)	0.05 (−1.1, 1.2)	0.93	0.09 (−1.1, 1.3)	0.88
Hypertension (reference: none)	0.2 (−1.2, 1.5)	0.81	0.05 (−1.3, 1.4)	0.94
Hyperlipidemia (reference: none)	0.1 (−1.1, 1.4)	0.83	0.1 (−1.1, 1.4)	0.83
Number of other medical conditions (per one condition) ^‡^	0.4 (−0.2, 0.9)	0.17	0.4 (−0.1, 0.9)	0.15
Use of anti-obesity medications (reference: no use before or after enrollment)				
Use before but not after enrollment	−1.9 (−4.1, 0.2)	0.08	−2.0 (−4.2, 0.2)	0.07
Use after but not before enrollment	−2.2 (−3.8, −0.7)	0.004	−2.3 (−3.8, −0.8)	0.003
Use before and after enrollment	−10.0 (−11.4, −8.7)	< 0.0001	−10.1 (−11.5, −8.7)	< 0.0001
**Engagement variables**				
Engagement with coach (reference: none)			0.3 (−1.5, 2.0)	0.74
Engagement with app (reference: none)			−0.2 (−2.2, 1.9)	0.86
Engagement with devices (reference: none)			1.0 (−0.5, 2.6)	0.18
Total months of engagement (0–6)			−0.6 (−0.9, −0.2)	0.005

CI = confidence interval

^*^From a multivariable linear regression model including all factors (except engagement variables) and adjusting for referring clinician using generalized estimating equations. Negative values indicate greater weight loss

^†^From a multivariable linear regression model including all factors plus engagement variables (categories of engagement and total months of engagement) and adjusting for referring clinician using generalized estimating equations. Negative values indicate greater weight loss

^‡^Ordinal (count) variable calculated by summing the total number of medical conditions for each patient, not including type 2 diabetes, prediabetes or insulin resistance, hypertension, and hyperlipidemia

## DISCUSSION

We rapidly enrolled a racially and ethnically diverse group of 5057 patients in PROPS 2.0 in less than one year. Patients lost weight during the first six months, with greater weight loss among those using anti-obesity medications. Registered patients lost more weight than non-registered patients, although this was non-significant in adjusted analyses. Longer duration of engagement, older age, and greater weight at enrollment were associated with greater weight loss.

These findings are consistent with previous studies showing that online weight management programs can help patients with weight loss.^9–11,18,19^ In the original PROPS Study, primary care patients who were randomized to an online program plus population health management lost significantly more weight compared to those randomized to an online program alone or to usual care.^15^ The amount of weight loss among patients in PROPS 2.0 was similar to the original PROPS Study, but PROPS 2.0 included a much larger and broader population of patients and had fewer inclusion/exclusion criteria.

To our knowledge, PROPS 2.0 is the largest project to date to integrate and evaluate an online weight management program in a diverse population of patients within a healthcare system. The recruitment and enrollment process utilized many features to increase scalability, including the referral order and BPAs within the EHR and the CRM platform to automate and manage tasks. The large number of referrals from clinicians (over 8000) and rapid enrollment demonstrate the high demand for this kind of program in clinical settings. Weight data were collected from digital scales that were synced with the online program and from the EHR, minimizing burden for patients and clinicians, and multiple imputation was used for missing data. Although patients who used anti-obesity medications experienced substantially greater weight loss than those who did not, the small differences between registered and non-registered patients in both groups suggest a potential added benefit of the program.

### Limitations

Some patients had difficulty completing the enrollment process electronically, using the online program, or syncing their devices, even though technical support was provided. Enrollment and registration rates were lower among patients who spoke Spanish as their primary language, even though we made some adjustments to try to address this.^16^ Furthermore, although the population was racially and ethnically diverse, most patients were female, college-educated, and had private insurance.

Given that PROPS 2.0 was an implementation project designed as a clinical program within standard care and offered to all eligible patients, there was no randomization or control group; thus, these data cannot prove that the program was effective. Although we compared weight change among patients who registered to those who did not, patients who did not register could have been participating in other programs outside of PROPS 2.0, which we were unable to assess, and this also could be true for patients who did register. Low rates of engagement are a likely explanation for the lack of a significant difference in weight loss between registered and non-registered patients. While over 90% of registered patients engaged during the first month, this decreased substantially over time, with only 34.5% engaging by month 6; these results are consistent with other studies of online weight management programs.^20–22^ Longer duration of engagement was associated with greater weight loss among registered patients, and patients who engaged for more than three months had significantly greater weight loss than those who did not register.

Another limitation is that the RestoreHealth program was provided at no cost to patients in PROPS 2.0, but appropriate reimbursement pathways will need to be established for the program to be sustainable in the future. The cost of the online program is approximately $750 per patient per year, and the cost of program support from patient navigators and/or similar staff at the healthcare system is between $100,000 and $200,000 per year; however, these numbers could vary based on the number of patients.

## CONCLUSIONS

We implemented an online weight management program as part of standard care and enrolled a large population of patients in less than one year. A high proportion of patients engaged in the program, and there was significant weight loss over six months. This program has the potential to be scaled and implemented at other institutions in the future.

## Supplementary Information

Below is the link to the electronic supplementary material.

Supplementary file1 (DOCX 40 KB)

## Declarations

### Prior Presentations

NA.
