# Supplementary material for: Engagement and Weight Change in an Online Weight Management Program in Clinical Settings: An Implementation Project
Source: J Gen Intern Med. 2026 Jun 3;41(13):3757–67. doi: 10.1007/s11606-026-10493-5 (PMC13631141; doi:10.1007/s11606-026-10493-5)
Supplement: Supplementary file 1 [file 11606_2026_10493_MOESM1_ESM.docx]

**APPENDICES**

**Supplement eTable 1. Choices for calculation of six-month weight change among enrolled patients**

| **Possible choices** | **Available data** | | | | | **Weights used to calculate weight change** | **No. (%) in each category** |
| --- | --- | --- | --- | --- | --- | --- | --- |
|  | **Baseline (-42 days to + 30 days)** | | | **Six months (+/- 60 days)** | |  |  |
|  | RestoreHealth  (RHB^*^) | Epic  (EPICB^†^) | Self-reported enrollment (EnrollB^‡^) | RestoreHealth (RH6^§^) | Epic (EPIC6*^‖^*) |  |  |
| **Choice 1** | Yes | NA | NA | Yes | NA | RH6 – RHB | 993 (19.6) |
| **Choice 2** | No | Yes | NA | NA | Yes | EPIC6 – EPICB | 2281 (45.1) |
| **Choice 3** | Yes | NA | NA | No | NA | Imp(RH6) – RHB | 238 (4.7) |
| **Choice 4** | No | Yes | NA | NA | No | Imp(EPIC6) – EPICB | 914 (18.1) |
| **Choice 5a**  **5b**  **5c**  **5d** | No | No | Yes | Yes  Yes  No  No | Yes  No  Yes  No | [{RH6 + EPIC6}/2] – EnrollB  [{RH6 + Imp(EPIC6)}/2] - EnrollB  [{Imp(RH6) + EPIC6}/2] - EnrollB  [{Imp(RH6) + Imp(EPIC6)}/2] - EnrollB | 22 (0.4)  10 (0.2)  351 (6.9)  247 (4.9) |

NA = not applicable (doesn’t matter whether present or not); Imp = imputed

^*^ Baseline weight from RestoreHealth (digital scale)

^†^ Baseline weight from Epic

^‡^ Baseline self-reported weight from enrollment survey

^§^ Six-month weight from RestoreHealth (digital scale)

*^‖^* Six-month weight from Epic

**Supplement eTable 2. Absolute weight change at six months, within four subgroups of use of anti-obesity medications**

|  | **No use before or after enrollment (n=3160)** | **Use before but not after enrollment (n=278)** | **Use after enrollment but not before (n=682)** | **Use before and after enrollment (n=936)** |
| --- | --- | --- | --- | --- |
| **Absolute weight change (pounds)** | | | | |
|  | **Unadjusted**  **mean (95% CI) *** | **Unadjusted**  **mean (95% CI) *** | **Unadjusted**  **mean (95% CI) *** | **Unadjusted**  **mean (95% CI) *** |
| All enrolled | -3.9 (-4.4, -3.5) | -4.6 (-6.5, -2.8) | -5.0 (-6.0, -3.9) | -12.5 (-13.5, -11.5) |
|  | **Adjusted**  **mean (95% CI) ^†^** | **Adjusted**  **mean (95% CI) ^†^** | **Adjusted**  **mean (95% CI) ^†^** | **Adjusted**  **mean (95% CI) ^†^** |
| Registered | -4.0 (-4.5, -3.5) | -4.8 (-6.6, -3.0) | -5.0 (-6.1, -4.0) | -12.7 (-14.0, -11.4) |
| Not registered | -3.2 (-5.0, -1.5) | -3.4 (-8.5, 1.7) | -4.4 (-7.7, -1.0) | -10.8 (-14.1, -7.5) |
| Difference | -0.8 (-2.6, 1.1) | -1.4 (-6.6, 3.8) | -0.7 (-4.1, 2.8) | -1.9 (-5.3, 1.5) |

CI = confidence interval

^*^ Unadjusted means and 95% CI from linear regression models (for absolute weight change and percent weight change) or a logistic regression model (for percent with ≥ 5% weight loss), adjusting for referring clinician using generalized estimating equations.

^†^ Adjusted means and 95% CI from linear regression models (for absolute weight change and percent weight change) or a logistic regression model (for percent with ≥ 5% weight loss), adjusting for referring clinician and age, sex, race, ethnicity, primary language, baseline weight, and medical conditions.

**Supplement eTable 3. Absolute weight change at six months among enrolled patients, by registration status and months of engagement**

|  |  | **Absolute weight change (pounds)** |
| --- | --- | --- |
| **Registration and months of engagement** | **No. (%)** | **Adjusted mean (95% CI) ^*^** |
| Registered, engaged > 3 months | 2042 (40.4) | -7.2 (-7.8, -6.5) ^†^ |
| Registered, engaged ≤ 3 months | 2469 (48.8) | -4.7 (-5.3, -4.1) ^‡^ |
| Not registered | 545 (10.8) | -4.7 (-6.2, -3.2) |

**^*^** Adjusted means and 95% CI from a linear regression model, adjusting for referring clinician and age, sex, race, ethnicity, primary language, baseline weight, medical conditions, and use of anti-obesity medications.

^†^ Difference between engaged > 3 months (vs. not registered) = -2.5 (95% CI, -4.1 to -0.9), P value = 0.003

^‡^ Difference between engaged ≤ 3 months (vs. not registered) = 0.01 (95% CI, -1.6 to 1.6), P value = 0.99

**Supplement eFigure 1.**

Number of Enrolled Patients

| Month | Clinician | Self | Electronic Health Record |
| --- | --- | --- | --- |
| 2022-11-01 | 81 | 16 | 0 |
| 2022-12-01 | 194 | 45 | 0 |
| 2023-01-01 | 421 | 73 | 0 |
| 2023-02-01 | 397 | 33 | 0 |
| 2023-03-01 | 533 | 66 | 0 |
| 2023-04-01 | 546 | 74 | 0 |
| 2023-05-01 | 654 | 50 | 0 |
| 2023-06-01 | 563 | 42 | 0 |
| 2023-07-01 | 343 | 36 | 49 |
| 2023-08-01 | 327 | 36 | 213 |
| 2023-09-01 | 127 | 15 | 58 |
| 2023-10-01 | 30 | 0 | 35 |
